# Magnesium Supplementation Shortens Hemodialysis-Associated Prolonged QT

**DOI:** 10.7759/cureus.9132

**Published:** 2020-07-11

**Authors:** Krishna Kishore Umapathi, Sunah Lee, Jessica Jacobson, Sara Jandeska, Hoang H Nguyen

**Affiliations:** 1 Pediatrics, Rush University Medical Center, Chicago, USA; 2 Pediatrics, Childrens Hospital of Los Angeles, Los Angeles, USA; 3 Pharmacology and Therapeutics, Rush University Medical Center, Chicago, USA; 4 Pediatric Nephrology, Rush University Medical Center, Chicago, USA; 5 Pediatrics, University of Texas Southwestern Medical Center, Dallas, USA

**Keywords:** magnesium, hemodialysis, chronic kidney disease, cardio protection, long qt

## Abstract

Hemodialysis affects myocardial depolarization and repolarization notably lengthening the QT interval. Prolonged QT, in turn, has been a reliable surrogate for higher risk of potentially lethal ventricular arrhythmias. We present an adolescent girl with end-stage kidney disease who consistently developed prolonged QT following hemodialysis sessions. Interestingly, her QT intervals were inversely correlated with her serum magnesium levels. Magnesium supplementation appeared to help reduce the QT prolongation after hemodialysis. Our case shows the potential utility of magnesium as a cardioprotective agent in hemodialysis patients. We recommend that patients undergoing hemodialysis receive frequent electrocardiograms and electrolytes monitoring for tailored electrolytes management to reduce the risk of developing potentially lethal cardiac arrhythmias.

## Introduction

QT interval prolongation has been widely reported in hemodialysis patients [1,2]. This surrogate of abnormal ventricular depolarization and repolarization, in turn, may trigger life-threatening ventricular arrhythmias [3,4]. Controversy exists as to the correlation between the concentration of serum and dialysate electrolytes on QT prolongation during and after dialysis [5-7]. We present a patient who developed new-onset QT prolongation and T wave abnormalities during and after hemodialysis associated with hypomagnesemia. QT interval and T wave abnormalities improved with magnesium supplementation; the degree of improvement was positively correlated with the level of serum magnesium. This case highlights the possible cardioprotective role of magnesium in restoring and maintaining myocardial electrical stability during and after hemodialysis.

## Case presentation

**Figure 1:**
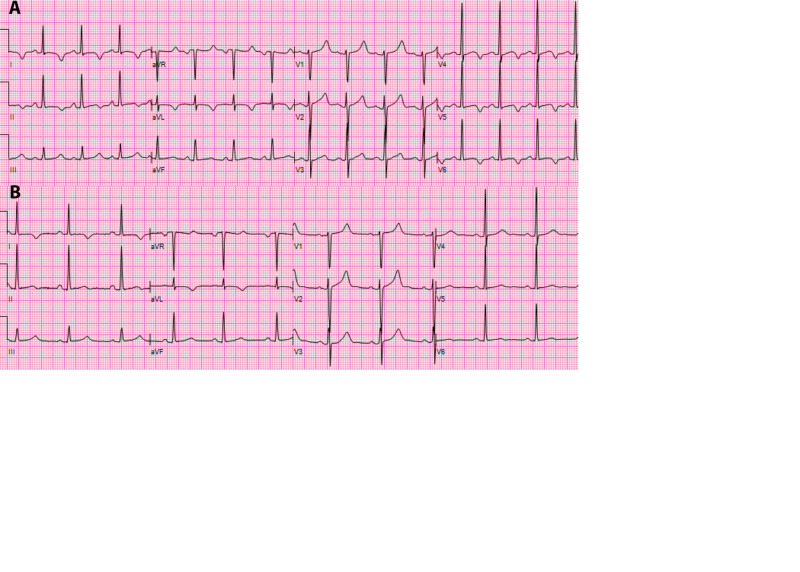
Electrocardiogram showing prolonged QT and T wave inversion in the lateral leads associated with hypomagnesemia (A) and shortened QT interval with upright normal T waves in the lateral precordial leads V5 and V6 when on magnesium supplementation (B)

**Figure 2:**
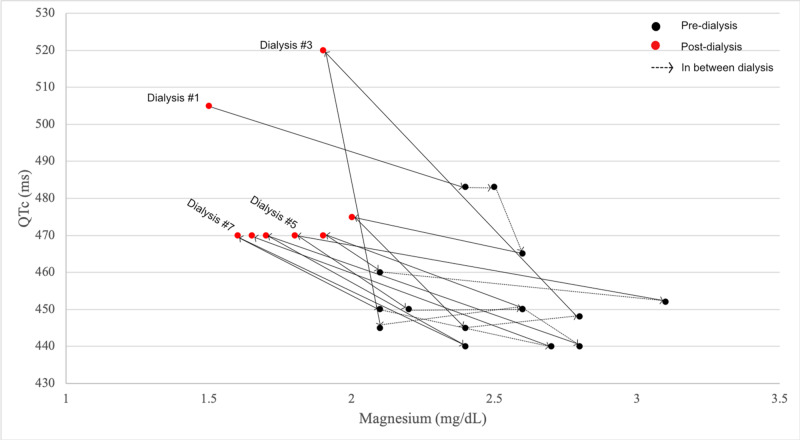
Connected scatter plot showing the association between QTc and magnesium across consecutive dialysis sessions. The data points are sequential in time. There is a pattern of decrease in magnesium and increase in QTc post-dialysis (red dots tend to be shifted upward and to the left). With initiation of scheduled magnesium supplementation (from dialysis #3 onward), there is less difference in magnesium and QTc pre- and post-dialysis sessions

A 19-year-old female with microscopic polyangiitis and resulting end-stage kidney disease was admitted for diarrhea and vomiting. She was found to have new-onset QT prolongation after a routine hemodialysis session. The prolonged QT (QTc = 485 ms) was incidentally found on electrocardiogram (ECG) obtained due to hyperkalemia. Worsening QT prolongation (QTc = 505 ms) and T wave inversion in the lateral leads were also observed on ECG after the next hemodialysis session two days later raising concerns for ischemia (Figure 1A). The troponin level at this time was normal and an echocardiogram showed normal systolic function with no regional wall motion abnormalities. The dialysate fluid utilized during hemodialysis sessions contained either 1.5% or 2.5% dextrose along with sodium 132 mEq/L, calcium 3.5 mEq/L, magnesium 0.5 mEq/L, chloride 96 mEq/L, and lactate 40 mEq/L. At the time of the abnormal ECG, the patient’s serum electrolytes were within normal limits except for hypomagnesemia (1.5 mg/dL). She received 2 grams IV magnesium supplementation post-dialysis raising the patient’s serum magnesium level to 2.4 mg/dL, corresponding with a QTc shortening to 483 ms. The patient was also started on a beta-blocker, nadolol, for management of prolonged QT syndrome, and all QT-prolonging medications (metronidazole, tacrolimus) were stopped. Thereafter, magnesium levels and ECGs were obtained before and after each dialysis session. We observed that the serum magnesium levels tended to decrease post-dialysis, with the inverse being true for QTc. Potassium and calcium serum levels (corrected for the slightly low levels of albumin) always remained within normal limits. With scheduled oral magnesium oxide supplementation following repeat dialysis sessions, these trends continued to be observed albeit at a much smaller amplitude (Figure 2). T wave abnormalities also improved with the scheduled magnesium supplementation, and no arrhythmias were observed during dialysis sessions (Figure 1B). Given the positive response to magnesium supplementation, the patient was discharged on oral magnesium oxide 500 mg twice daily supplementation with close cardiology follow-up.

## Discussion

Chronic hemodialysis has been associated with abnormal cardiac depolarization and repolarization manifested as QT prolongation, increased QT dispersion, and reduced capacity of the QT interval to adapt to heart rate changes [8]. The prevalence of QT interval prolongation has been reported between 39% and 47% of chronic hemodialysis patients [9,10]. QT prolongation has been associated with increased risk of lethal arrhythmias such as torsades des pointes. The exact mechanism of QT prolongation in end-stage renal disease patients is likely to be multi-factorial. For example, some studies have postulated that QT prolongation is secondary to left ventricular hypertrophy and fibrosis while others point to the rapid shifts of electrolytes in and out of the cardiac myocytes during hemolysis as the cause of prolonged QT [11,12].

Blood levels of electrolytes such as potassium and calcium have been shown to affect QT duration in healthy people, and these findings have been extended to the hemodialysis patients [7,13-17]. Specifically, the percentage reduction in serum potassium was significantly higher in patients with a post-dialysis increase in QTc interval duration as compared to those with a post-dialysis decrease in QTc [6]. On the other hand, low serum calcium during hemodialysis can cause QT prolongation by lengthening left ventricular relaxation [5].

Intuitively, low concentrations of potassium and calcium in the dialysis may also affect the QT interval [1]. This may be because the gradient between the blood and dialysate concentrations of these electrolytes may also be important in maintaining the electrical stability of the cardiac myocytes. However, tighter control of the dialysis bath composition with the resultant stable plasma electrolytes concentrations during and after the hemodialysis sessions did not decrease the prevalence of QTc interval prolongation suggesting that other risk factors such as cardiac fibrosis and calcification may also play a role in causing the abnormal alterations of ventricular repolarization [18].

Other dialysis-related factors including ultrafiltration rates and dialysis times have been proposed to play a role in the development of QTc prolongation. A high ultrafiltration rate has been linked to myocardial stunning and delayed cardiac recovery following dialysis, both of which may be reflected in the prolonged QT interval [19]. On the other hand, a longer dialysis time, as seen with nocturnal dialysis sessions, has been reported to be associated with shorter QT as a longer hemodialysis time is hypothesized to be necessary to adequately remove a cardio-toxic substance to ventricular depolarization [20].

While the association between low calcium and low potassium levels with the development of prolonged QT intervals appears to be well established, controversy remains in regard to the role serum magnesium levels in lengthening the QT interval [5]. In the general healthy population, higher serum magnesium levels were associated with longer QT. However, the effect size of magnesium was fairly small. In hemodialysis patients, the relationship between low serum magnesium and prolonged QT was reported in some studies while not confirmed in others [1,2,6]. The association between low magnesium and long QT has been reported in other patient populations such as chronic alcoholics. Chronic alcoholics were shown to be nine times more likely to have long QT and three times more likely to have low magnesium than abstinent alcoholics.

The prolongation of the ventricular repolarization period can allow for early after-depolarization to induce the development of tachyarrhythmia, specifically torsades de pointes. Additionally, it has been shown that the risk of torsades des pointes increases two- to three-fold with QTc of > 500 ms. Interestingly, magnesium suppresses early after depolarization, likely by correcting the inadequate change of frequencies in the repolarization period, thus reducing the variation of the QT interval. This suppression could explain why intravenous magnesium infusions revert long QT-triggered arrhythmias. Intravenous magnesium can suppress episodes of torsades des pointes without necessarily shortening QT. This effect can take place even if the levels of magnesium are normal. In psychiatric patients with prolonged QT, oral magnesium supplementation was shown to effectively shorten the QTc allowing patients to be maintained on QTc prolonging antipsychotic medications. Magnesium could also reduce certain types of inadequate kinetics of rate adaptation of the QT. Magnesium itself does not influence the rate-dependent adaptation of the repolarization period. However, magnesium is a co-factor of sodium-potassium ATPase activity. By facilitating the influx of potassium into the cells it stabilizes membrane potential, correcting the inadequate rate-dependent repolarization process.

Initiation of a beta-blocker is indicated in cases of congenital long QT as they have the potential to reduce the length of the QT interval and improves prognosis. However, in this patient, QT prolongation appeared dynamic and associated with hemodialysis and level of magnesium despite treatment with the beta-blocker nadolol. Moreover, our patient did not appear to have cardiac risk factors such as left ventricular hypertrophy or dilated cardiomyopathy. Finally, her serum calcium and potassium levels remained within normal limits throughout dialysis sessions without large gradients being seen pre- and post-dialysis. Serum magnesium of >2.1 mEq/L has been shown to be associated with better survival among hemodialysis patients in the United States. We, therefore, surmise that the QT prolongation was more likely associated with the fluctuation in serum magnesium levels.

## Conclusions

Magnesium supplement to maintain normal to the high normal serum level of magnesium appear to minimize the QT prolongation post hemodialysis, making this a potentially modifiable risk factor and effective means for decreasing the risk of cardiac arrhythmias. Additional investigation is needed to determine the exact role and mechanism by which magnesium supplementation may benefit chronic hemodialysis patients and the potential reproducibility of the treatment outcome. In the meantime, we believe that the dialysis regimen must be personalized to each patient. Routine ECG monitoring, along with serum electrolyte monitoring (calcium, potassium, and magnesium) for patients on hemodialysis should be obtained following each dialysis session to identify patients with abnormal ventricular repolarizations. In these patients, we can tailor a dialysis regimen aimed to less likely affect ventricular repolarization.
